# Supplementary material for: Cloud-native simulation framework for gossip protocol: Modeling and analyzing network dynamics
Source: PLoS One. 2025 Jun 23;20(6):e0325817. doi: 10.1371/journal.pone.0325817 (PMC12184886; doi:10.1371/journal.pone.0325817)
Supplement: Attachment — Submitted filename: Response to Reviewers.docx [file pone.0325817.s001.docx]

**Manuscript ID: PONE-D-24-43101**

**Original Article Title:** Cloud-Native Simulation Framework for Gossip Protocol: Modeling and Analyzing Network Dynamics

**To:** Academic Editor, PLOS ONE

**Re:** Response to editor and reviewers

Dear Editor,

Thank you for allowing a resubmission of our manuscript, with an opportunity to address the editor’s and reviewers’ comments.

We are uploading the following three files

(a) the response letter to the comments of the academic editor and reviewers;

(b) the revised paper with track change;

(c) a clean revised paper.

Best regards,

Samsuddin Wira

**Editor**

**We would like to thank the editor for the guidance and constructive comments in improving the quality and readability of this manuscript according to PLOS ONE’s publication standard. The effort and time in reviewing this paper are highly appreciated. We have rigorously revised the manuscript based on your comments and suggestions as follows:**

**Comment 1:** Please ensure that your manuscript meets PLOS ONE's style requirements, including those for file naming. The PLOS ONE style templates can be found at

https://journals.plos.org/plosone/s/file?id=wjVg/PLOSOne_formatting_sample_main_body.pdf and

https://journals.plos.org/plosone/s/file?id=ba62/PLOSOne_formatting_sample_title_authors_affiliations.pdf.

**Author response:** In response to Comment 1 regarding PLOS ONE's style requirements, we confirm our manuscript now fully complies with all guidelines, including file naming, as per the provided templates.

The revised format and guidelines are as follows:

| **Amendment** | **Page** | **Section** |
| --- | --- | --- |
| Original: Authors without roles  Amended: Authors with roles | 1 | Introduction |
| Original: Fig 1 with no PACE tool image converter  Amended: Fig 1 with PACE tool image converter | 2 | Introduction |
| Original: Fig 2 with no PACE tool image converter  Amended: Fig 2 with PACE tool image converter | 10 | Simulation Design and Framework Selection |
| Original: Fig 3 to Fig 6 with no PACE tool image converter  Amended: Fig 3 to Fig 6 with PACE tool image converter | 14 - 15 | Results |

**Comment 2:** Please note that PLOS ONE has specific guidelines on code sharing for submissions in which author-generated code underpins the findings in the manuscript. In these cases, all author-generated code must be made available without restrictions upon publication of the work. Please review our guidelines at https://journals.plos.org/plosone/s/materials-and-software-sharing#loc-sharing-code and ensure that your code is shared in a way that follows best practice and facilitates reproducibility and reuse.

**Author response:** We thank the editor for highlighting PLOS ONE’s code-sharing guidelines. We fully support the journal’s commitment to transparency, reproducibility, and the open sharing of research materials. In line with these guidelines, we have made all author-generated code underlying the findings of our manuscript publicly available without restrictions.

A public repository [<https://github.com/wwiras/cnsim.git>] has been created and is accessible at the following link, which will be included in the manuscript.

The repository includes:

- Source Code: All code used for the simulations, modeling, and analysis presented in the manuscript.
- Documentation: Detailed instructions for setting up, running, and reproducing the results.
- License: An open-source license to facilitate reuse and further development by the research community.

We have carefully reviewed and followed PLOS ONE’s best practices for sharing code, as outlined at https://journals.plos.org/plosone/s/materials-and-software-sharing#loc-sharing-code. We believe this will enhance the reproducibility of our work and encourage its reuse in future studies. Please let us know if any additional steps are required to ensure full compliance with the journal’s guidelines.

**Comment 3:** In your Methods section, please include additional information about your dataset and ensure that you have included a statement specifying whether the collection and analysis method complied with the terms and conditions for the source of the data.

**Author response:** We thank the editor for this important comment. In response, we have revised the Methods section of our manuscript to include additional details about the dataset used in this study. Specifically, we have added the following information:

| **Amendment** | **Page** | **Section** |
| --- | --- | --- |
| Original:  The steps taken to implement a gossip protocol in a cloud-native simulation are as follows:-  Amended:  The following steps outline the gossip protocol implementation for this cloud-native simulation. All research artifacts—including synthetically generated datasets and the complete source code—are publicly available at https://github.com/wwiras/cnsim.git. This work complies with all relevant ethical and legal guidelines, and no external restrictions apply to its data or methodology. | 12 | Steps for Implementing Gossip Protocol in a Cloud Native Simulation |

**Comment 4:** Thank you for stating the following financial disclosure:

“Internal Matching Grant - Public Service Department (JPA) of Malaysia”

Please state what role the funders took in the study. If the funders had no role, please state: "The funders had no role in study design, data collection and analysis, decision to publish, or preparation of the manuscript."

If this statement is not correct you must amend it as needed.

Please include this amended Role of Funder statement in your cover letter; we will change the online submission form on your behalf.

**Author response:** We thank the editor for bringing this to our attention. This work was funded through a joint initiative of the Public Service Department (JPA) of Malaysia and a Monash Internal Matching Grant from the School of Information Technology, Monash University Malaysia. However, the funders had no role in the study design, data collection and analysis, decision to publish, or preparation of the manuscript.

We have amended the Role of Funder statement in the cover letter as follows:

Amended Role of Funder Statement:

"The funders had no role in study design, data collection and analysis, decision to publish, or preparation of the manuscript."

Please update the online submission form on our behalf to reflect this statement. Thank you for your assistance.

**Comment 5:** Thank you for stating the following in the Acknowledgments Section of your manuscript:

“This work is supported by Monash University Malaysia through an internal matching

grant, in collaboration with the Public Service Department (JPA) of Malaysia .”

We note that you have provided funding information that is currently declared in your Funding Statement. However, funding information should not appear in the Acknowledgments section or other areas of your manuscript. We will only publish funding information present in the Funding Statement section of the online submission form.

Please remove any funding-related text from the manuscript and let us know how you would like to update your Funding Statement. Currently, your Funding Statement reads as follows:

“Internal Matching Grant - Public Service Department (JPA) of Malaysia”

Please include your amended statements within your cover letter; we will change the online submission form on your behalf.

**Author response:** We thank the editor for pointing out the duplication of funding information in the Acknowledgments section. We have removed the following sentence from the Acknowledgments section of the manuscript:

“This work is supported by the School of Information Technology, Monash University Malaysia through an internal matching grant, in collaboration with the Public Service Department (JPA) of Malaysia.”

We confirm that the funding information will now only appear in the Funding Statement section of the online submission form. Please update the Funding Statement to the following:

| **Amendment** | **Page** | **Section** |
| --- | --- | --- |
| Funding-related text is removed from the manuscript in the “Acknowledgement Section”. | 21 | Acknowledgement |

**Comment 6:** In the online submission form, you indicated that [Data available on request from the author through email.].

All PLOS journals now require all data underlying the findings described in their manuscript to be freely available to other researchers, either 1. In a public repository, 2. Within the manuscript itself, or 3. Uploaded as supplementary information.

This policy applies to all data except where public deposition would breach compliance with the protocol approved by your research ethics board. If your data cannot be made publicly available for ethical or legal reasons (e.g., public availability would compromise patient privacy), please explain your reasons on resubmission and your exemption request will be escalated for approval.

**Author response:** We thank the editor for bringing this to our attention. Following PLOS ONE’s data-sharing policy, we confirm that all data underlying the findings described in our manuscript will be made publicly available to all researchers.

We have changed the data availability from “available on request” to publicly available. The dataset used in this study has been deposited in a public repository and is accessible at the following link:

<https://github.com/wwiras/cnsim.git>

The repository includes:

1. Source Data: All data used for the simulations, modeling, and analysis are presented in the manuscript.
2. Documentation: Detailed instructions for accessing and using the data.
3. License: An open-source license to facilitate reuse and further research.

Please let us know if further clarification or additional steps are required. Thank you for your guidance in ensuring compliance with PLOS ONE’s policies.

**End**

**Reviewer 1**

**We would like to take this opportunity to thank the reviewers for their constructive comments and suggestions in improving the quality and readability of this manuscript. Your effort and time in reviewing this paper are highly appreciated. We have rigorously revised the manuscript based on the reviewers’ comments as follows:**

**Comment 1:** The figures are not visible in the manuscript, this gives the impression that the submission is a draft. Moreover, this makes it very hard to verify the results.

**Author response:** We sincerely thank the reviewer for pointing out the issue with the figures in our manuscript. We apologize for the inconvenience caused by the poor visibility of the figures. We have taken the following steps to address this concern. All figures have been revised to ensure high resolution and sharpness using the PLOS ONE PACE tool (<https://pacev2.apexcovantage.com/>). This ensures all figures follow PLOS ONE guidelines. We believe these changes will significantly improve the reader’s ability to verify the results and enhance the overall quality of the manuscript. Thank you again for your valuable feedback, which has helped us improve the presentation of our work.

The revised figures are as follows:

| **Amendment** | **Page** | **Section** |
| --- | --- | --- |
| Original: Fig 1 with no PACE tool image converter  Amended: Fig 1 with PACE tool image converter | 2 | Introduction |
| Original: Fig 2 with no PACE tool image converter  Amended: Fig 2 with PACE tool image converter | 10 | Simulation Design and Framework Selection |
| Original: Fig 3 to Fig 6 with no PACE tool image converter  Amended: Fig 3 to Fig 6 with PACE tool image converter | 14 - 15 | Results |

**Comment 2:** The insights and key findings reported in the conclusion seem trivial and could be easily expected.

**Author response:** We sincerely thank you for the feedback. Some insights and key findings in the conclusion may appear trivial or expected. However, these results are valuable when considering the broader context of gossip protocol simulation in cloud-native environments, particularly when multiple factors or parameters are analyzed in a comprehensive manner.

In our opinion, the value of this work lies in its application to cloud-native systems, which offer several advantages over conventional simulators or bare-metal setups:

1. Flexibility: In cloud-native environments, resources such as CPU, memory, and network are elastic, allowing for dynamic scaling based on experimental needs. In contrast, bare-metal setups or traditional simulators (e.g., PeerSim, Mininet) have limited resource availability and unused resources often go to waste after the experiment.
2. Cost Efficiency: Cloud-native platforms enable pay-as-you-go models, where users only pay for the resources consumed during the experiment. This is a critical factor for large-scale simulations, as it significantly reduces costs compared to maintaining dedicated hardware or using specialized simulation tools.
3. Comprehensive Analysis or Observability: While traditional simulators like PeerSim or Mininet primarily focus on network-level analysis, cloud-native environments allow for evaluating additional factors such as CPU and memory usage, which are crucial in modern distributed systems like blockchain. This holistic approach provides deeper insights into the performance and scalability of gossip protocols under realistic conditions.

Combining these benefits makes cloud-native simulation a powerful tool for studying gossip protocols, even if some of the individual results may seem expected. Our work lays the foundation for future research, where more advanced factors (e.g., clustering, network topology) can be explored in a cost-effective and scalable manner.

Thank you for your comment, which has allowed us to better articulate the significance of our findings in the context of cloud-native systems.

To better communicate the impact of our research, the subsection of Contributions and Significance of the Study (under the Conclusion section) has been rewritten to highlight the main contributions and findings.

| **Amendment** | **Page** | **Section** |
| --- | --- | --- |
| Original:  This study presents a detailed examination of gossip protocols within cloud-native environments, significantly contributing to cloud-native computing and distributed systems. The research highlights the challenges with the scalability and performance of gossip protocols as the network size increases, providing a nuanced understanding of message propagation dynamics across varying network scales. This aspect is particularly significant for cloud-native systems, emphasizing the need for adaptable and scalable communication mechanisms to handle rapid network size changes without compromising efficiency.  The study also provides valuable insights into optimization strategies for implementing gossip protocols in cloud-native applications. By presenting concrete metrics on the impact of node quantity on protocol performance, the research validates theoretical expectations regarding scalability challenges. It proposes practical solutions for enhancing system reliability and message propagation speed. The findings of this research are highly relevant for industries that rely on robust, scalable, and efficient communication mechanisms, such as blockchain, IoT, and mobile applications. The study suggests tailored adjustments to gossip protocol configurations can significantly improve performance across diverse cloud-native settings.  Moreover, the study emphasizes gossip protocols' broader applicability and potential to advance distributed systems' resilience, scalability, and efficiency. By elucidating the protocol's dynamics within cloud-native environments, the research opens opportunities for more adaptable and robust distributed architectures, offering substantial theoretical and practical advancements. Consequently, this study not only enriches the existing body of knowledge on gossip protocols and cloud-native computing but also sets the stage for future innovations in deploying and optimizing distributed systems across various sectors.  Amended: This study contributes to understanding gossip protocols in cloud-native environments by empirically validating theoretical assumptions and revealing infrastructure-specific behaviors. While some scalability patterns confirm established knowledge (like propagation delays at scale), the research demonstrates how elastic, pay-as-you-go cloud platforms fundamentally alter protocol dynamics - notably showing that 30Mbps bandwidth reduces CPU usage by 40% while increasing memory consumption by 15-20%, and that 300Mi memory allocations achieve 25% greater CPU efficiency than traditional 150Mi configurations. These quantified trade-offs, observable only in cloud- native, provide actionable optimization guidelines for blockchain and IoT deployments. The work establishes three key advances:  • a cloud-native methodology capturing multidimensional metrics (CPU, memory, network) simultaneously, overcoming limitations of conventional simulators like PeerSim;  • identified configuration thresholds that enable practical performance tuning; and  • a reproducible framework for future research on advanced factors like topology-aware propagation without dedicated hardware costs.  The significance of these findings lies in their practical applicability and methodological innovation. By bridging theoretical protocol behavior with cloud-native constraints, the research demonstrates that expected patterns manifest differently in elastic infrastructures - for instance, the observed 40% CPU reduction at 30Mbps incurs memory costs unique to dynamic environments. This work provides practitioners with evidence-based strategies for optimizing distributed systems, while offering researchers a cost-effective experimental platform to investigate scalability-resource trade-offs. The results not only advance gossip protocol implementation in production environments but also establish foundational knowledge for developing more resilient and adaptive cloud-native architectures. | 19 - 20 | Contributions and Significance of the Study |

**Comment 3:** Considering the additional network traffic that one could expect when employing gossip protocols, there are serious doubts on the feasibility of using gossip protocols in real-world cloud deployments. This is mostly because currently one of the biggest cost factors is due to the network traffic between availability zones and regions (needed for fault tolerance and high availability), especially in highly distributed cloud native scenarios using microservices architecture. Performance/scalability is relevant, but efficiency can be nowadays even more important. In my opinion, the related work section should focus in relating gossip protocols to other communication patterns suitable to cloud native architectures, instead of comparing to other simulators.

**Author response:** We sincerely thank the reviewer for their insightful comment regarding the feasibility of gossip protocols in real-world cloud deployments and the suggestion to refocus the related work section. We agree that network traffic costs, particularly between availability zones and regions, are a critical factor in cloud-native architectures, and efficiency is often as important as performance and scalability.

In response to this comment, we have revised the Related Work section to focus on relating gossip protocols to other communication patterns suitable for cloud-native architectures, rather than comparing them to other simulators. This additional information is put in Table 1. Thank you for your valuable suggestion, which has significantly improved the depth and relevance of the manuscript.

The revised statement reads as follows:

| **Amendment** | **Page** | **Section** |
| --- | --- | --- |
| Original:  Table 1. Summary of Well-Known Gossip Protocols  Amended:  Table 1. Gossip Protocols and their Relation to Cloud Native Architecture. | 25 | Background and Related Works |

**Comment 4:** Limited contributions. It seems hard to see how the claim on contribution 1 (Development of an Adaptive Simulator) that such a tool would provide on “translating theoretical concepts of gossip protocols into practical, actionable insights”. Simulators are valid tools, but we are used to expecting the threats to validity about their practical implications. Moreover, it seems difficult to the claimed contribution 2 ( a tutorial) as a scientific contribution.

**Author response:** We sincerely appreciate the reviewer's valuable feedback regarding the objectives and its contributions. The original framing made the practical implications appear somewhat limited. In response, we have significantly revised both the tutorial component and overall contributions to highlight their scientific value better. The tutorial has been reframed as a methodological framework for reproducible cloud-native experimentation, emphasizing standardized configuration templates, multi-metric observability, and empirically derived resource optimization profiles.

For the adaptive simulator contribution, we have strengthened the connection between theoretical concepts and practical insights by adding comprehensive observability metrics (CPU, memory, network) and explicitly discussing validity threats. The revised contributions now more clearly demonstrate how this work advances gossip protocol research through (1) enabling hardware-independent, reproducible experimentation, (2) providing quantifiable performance trade-offs (e.g., the 40% CPU reduction at 30Mbps bandwidth), and (3) establishing optimization guidelines for real-world deployment. These revisions better articulate the work's novel contributions to research methodology and practical implementation in cloud-native environments.

To respond to this point, the revised statements are as follows:

| **Amendment** | **Page** | **Section** |
| --- | --- | --- |
| Original:  Development of an Adaptive Simulator: An essential contribution of this research is the creation of a simulator designed specifically for gossip protocol development. This simulator offers the flexibility to thoroughly analyze gossip protocols' performance across different computational settings, focusing on scalability, fault tolerance, and adaptability. This tool is a step forward in translating theoretical concepts of gossip protocols into practical, actionable insights.  Amended:  Development of an Adaptive Simulator: An essential contribution of this research is the creation of a simulator designed specifically for gossip protocol development. This simulator offers the flexibility to extensively analyze gossip protocols' performance through a comprehensive analysis of scalability, fault tolerance, and resource efficiency (CPU/memory/network) across diverse cloud-native environments, contributing empirical insights for protocol optimization. | 5 | Introduction |
| Original:  A Comprehensive Tutorial on Modelling Framework and Simulator Usage: Recognizing the importance of accessibility and practical application, we provide a comprehensive tutorial on using the newly developed simulator. This guide is crafted to assist newcomers and seasoned practitioners in effectively utilizing the simulator to develop, test, and refine gossip protocols. It is an essential resource for anyone interested in exploring the nuances of gossip protocol performance.  Amended:  A Methodological Framework for Reproducible Cloud-Native Experimentation: This study establishes structured methods for evaluating gossip protocol performance, enabling reproducible investigation of dynamic scaling and topology sensitivity using cloud-native capabilities for future and extended research. | 5 | Introduction |

**Comment 5:** Considering that the research had access to a reasonable setup (Table 4) from Google Kubernetes Engine with 56 CPUs, 96 GB of memory etc, I wonder why the gossip protocol and the deployment still had to be simulated. How far are we from having actual implementations and real-world experiments evaluating the gossip protocols feasibility? Currently, using the abstractions and automations from cloud native architectures should make it possible in a productive way to have realistic test scenarios. In the past, we used simulations mostly because the access to suitable machines was complicated.

**Author response:** We sincerely thank the reviewer for their insightful comment regarding the use of a simulator despite having access to a real cloud-native environment like Google Kubernetes Engine (GKE). We agree that cloud-native architectures, with their abstractions and automation, provide a powerful platform for realistic test scenarios. However, we believe that simulation remains a critical tool for several reasons, even when real-world environments are available:

1. Controlled Experimentation:

While real-world environments like GKE are excellent for deployment and testing, they are inherently difficult to control due to external factors such as network variability, resource contention, and unpredictable workloads. A simulator allows us to precisely control variables (e.g., network latency, packet loss, CPU/memory constraints) and isolate their impact on the gossip protocol’s performance. This level of control is essential for validating theoretical models and understanding the protocol’s behavior under specific conditions. In addition, we add observability metrics (CPU usage, memory utilization, and network bytes transmitted/received) to get more insights for the gossip test.

2. Cost and Scalability:

Real-world experiments at scale (e.g., hundreds or thousands of nodes) can be prohibitively expensive due to the costs of cloud resources. Simulators enable us to test large-scale scenarios in a cost-effective manner without requiring extensive cloud infrastructure. Additionally, simulators allow us to explore edge cases and extreme scenarios that may be difficult or impractical to replicate in a real environment.

3. Validation Before Real-World Deployment:

Before deploying a gossip protocol in a real-world environment, validating its feasibility and performance in a controlled setting is crucial. A simulator provides a safe and reproducible environment for this purpose, ensuring that the protocol behaves as expected before being subjected to the complexities of a real-world deployment.

4. Flexibility and Customization:

Simulators can be customized and adapted to model various scenarios, including those that existing cloud-native platforms may not yet support. This flexibility is particularly valuable for exploring novel architectures or emerging use cases.

In summary, while real-world environments like GKE are invaluable for testing and deployment, simulators are complementary by providing controlled, scalable, and cost-effective experimentation. They enable us to validate theoretical concepts, optimize protocols, and explore new ideas before committing to real-world implementation.

Thank you for your comment, which has allowed us to better articulate the rationale behind using a simulator in this study.

To respond to this point, we updated our simulator factors. The revised statement reads as follows:

| **Amendment** | **Page** | **Section** |
| --- | --- | --- |
| Amended (Additional):  This study investigates the impact of network and hardware configurations on gossip protocol performance in Kubernetes clusters. We analyze the relationship between node count and gossip propagation time using comparative line graphs to characterize scaling behavior. Our simulation framework monitors three key dimensions:   - Network throughput (transmitted/received bytes) - Computational load (CPU utilization) - Memory allocation patterns   This multi-layer analysis reveals system-level performance trends (propagation latency scaling) and component-level resource dynamics, providing actionable insights for optimizing gossip protocols in production cloud environments. | 12 | Parameters and Variables for Gossip Protocol Simulation |

**End**

**Reviewer 2**

**We would like to thank the reviewers for their constructive comments and suggestions in improving the quality and readability of this manuscript. Your effort and time in reviewing this paper are highly appreciated. We have rigorously revised the manuscript based on the reviewers’ comments as follows:**

**Comment 1:** The manuscript presents a technically sound study exploring the implementation of gossip protocols within cloud-native frameworks. The experiments are rigorously conducted using Google Kubernetes Engine (GKE), and the methodology is detailed, including data creation, collection, and monitoring. The results are clearly presented using histograms and graphs, effectively supporting the conclusions about scalability, resilience, and optimization opportunities for gossip protocols. However, the scalability test is limited to 400 nodes, which may not fully represent large-scale cloud-native environments. Expanding this scope would enhance the validity of the conclusions.

**Author response:** We sincerely thank you for the positive feedback on the technical soundness of the study and the clarity of the results. We appreciate the suggestion to expand the scalability test to better represent large-scale cloud-native environments.

In response to this comment, we have increased the maximum number of nodes to 600 in our experiments. Additional resources are added to meet this requirement. This represents the maximum resources available to us at the moment of this writing, and we believe it provides a more robust evaluation of the gossip protocol’s scalability. The simulator's design, however, does not impose a theoretical limit on node count.

In future experiments, depending on resource availability, we aim to scale the number of nodes to 1000 or beyond. While the current study focuses on the foundational aspects of gossip protocols in cloud-native environments, expanding the scalability scope will further enhance the validity of the conclusions. The current results, combined with our plans, provide a strong foundation for ongoing research in this area.

Thank you for your valuable suggestion, which has helped us improve the manuscript and outline a clear direction for future work.

The revised number of nodes is as follows:

| **Amendment** | **Page** | **Section** |
| --- | --- | --- |
| Additional resources for the simulator  Original: Table 4 with with 24 nodes, 48 vCPUS and 96GB of memory  Amended: Table 4 with 36 nodes, 72 vCPUS and 144GB of memory | 27 | Simulation Design and Framework Selection |
| Original : Fig4, Fig5 and Fig6 with 400 nodes  Amended: Fig4, Fig5 and Fig6 with 500 to 600 nodes | 15 | Results |
| Bandwidth Adjustment Gossip Test  Original:  Analyzing the propagation times in the gossip protocol across various node configurations, as shown in Fig 4, reveals a clear pattern influenced by bandwidth variations. The graph shows a linear increase in propagation times as the number of nodes escalates, with the 5Mbps bandwidth setting resulting in the most extended times across the spectrum. Significantly, the 30Mbps configuration maintains markedly lower propagation times than the 5Mbps setting and the default scenario. This difference becomes more pronounced as the number of nodes increases, particularly noticeable from 30 nodes onwards, confirming that higher bandwidth significantly enhances the efficiency of gossip propagation in more extensive networks.  Amended:  Analyzing the propagation times in the gossip protocol across various node configurations, as shown in Fig 4, reveals a clear pattern influenced by bandwidth variations. The graph shows a linear increase in propagation times as the number of nodes escalates, with the 5Mbps bandwidth setting resulting in the most extended times across the spectrum. Significantly, the 30Mbps configuration maintains markedly lower propagation times than the 5Mbps setting. This difference becomes more pronounced as the number of nodes increases, particularly noticeable from 200 nodes onwards, confirming that higher bandwidth significantly enhances the efficiency of gossip propagation in more extensive networks. | 14 - 15 | Results |
| Memory Adjustment Gossip Test  Original:  Fig 5 illustrates the propagation times for gossip protocols across various node counts in a Kubernetes environment, specifically comparing setups with 150Mi and 300Mi memory allocations against a default configuration. All three configurations exhibit similar propagation times for networks with fewer than 100 nodes. However, as the node count increases beyond this point, the impact of increased memory allocation becomes more apparent. The propagation time in the 300Mi setup is consistently lower than that in the 150Mi setup across larger node counts, indicating improved performance. However, as the network size reaches 300 nodes, the differences in propagation times between the configurations begin to converge, suggesting diminishing returns on increased memory allocation at higher node scales.  Amended:  Fig 5 illustrates the propagation times for gossip protocols across various node counts in a Kubernetes environment, specifically comparing setups with 150Mi and 300Mi memory allocations against a default configuration. All three configurations exhibit similar propagation times for networks with fewer than 100 nodes. However, as the node count increases beyond this point, the impact of increased memory allocation becomes more apparent. The propagation time in the 300Mi setup is consistently lower than that in the 150Mi setup across larger node counts, indicating improved performance. However, as the network size reaches 400 nodes, the differences in propagation times between the configurations begin to converge, suggesting diminishing returns on increased memory allocation at higher node scales. | 15 | Result |
| Centralized (Single Zone) and Distributed (Multi-Zones) Setup Gossip Test  Original:  Fig 6 depicts the millisecond propagation times for gossip protocols across Kubernetes node-setups ranging from 10 to 400 nodes under two configurations: single zone and multi-zones. In the single-zone configuration, all nodes are located within the same geographical zone, while in the multi-zone configuration, nodes are distributed across different geographical zones. The data reveals that propagation times increase as the number of nodes grows. Notably, there is a consistent divergence in propagation times between the single-zone and multi-zone configurations starting from 70 nodes, indicating that geographical distribution has a considerable impact on the efficiency of the gossip protocol.  Amended:  Fig 6 depicts the millisecond propagation times for gossip protocols across Kubernetes node-setups ranging from 10 to 600 nodes under two configurations: single zone and multi-zones. In the single-zone configuration, all nodes are located within the same geographical zone, while in the multi-zone configuration, nodes are distributed across different geographical zones. The data reveals that propagation times increase as the number of nodes grows. Notably, there is a consistent divergence in propagation times between the single-zone and multi-zone configurations starting from 300 nodes, indicating that geographical distribution has a considerable impact on the efficiency of the gossip protocol. | 15 | Result |

**Comment 2:** The statistical analysis is appropriate, with results presented in a clear and interpretable manner. Propagation times and other performance metrics are analyzed across different node configurations and network setups, providing meaningful insights into the behavior of gossip protocols. The use of distribution histograms and line graphs effectively illustrates the patterns observed. However, incorporating additional metrics (e.g., CPU usage, network latency) could further enrich the analysis.

**Author response:** We sincerely thank the reviewer for the positive feedback on the statistical analysis and presentation of results. We appreciate the suggestion to incorporate additional metrics to further enrich the analysis.

In response to this comment, we have added the recommended features highlighted by the reviewer:

CPU Usage: We have included CPU usage metrics across different node configurations and network setups to evaluate the computational load imposed by the gossip protocol.

Memory Usage: We have added memory usage metrics to assess the impact of the gossip protocol on system resources under varying conditions.

Network Packets Sent/Received: We have included metrics for network activity, specifically tracking the number of packets sent and received, to analyze the communication overhead and efficiency of the gossip protocol.

These additional metrics have been integrated into the revised manuscript, and the results are presented using distribution histograms and line graphs to maintain consistency with the existing analysis. As a result, these enhancements provide deeper insights into the behavior of gossip protocols and further strengthen the manuscript. The “latency” evaluation aspect in our abstract is replaced by “observability” for a more meaningful definition and capabilities.

The amendments are as follows:

| **Amendment** | **Page** | **Section** |
| --- | --- | --- |
| Original:  The simulation analysis provides insights into gossip protocols’ behavior in distributed cloud-native systems, evaluating aspects of scalability, reliability, and latency.  Amended:  The simulation analysis provides insights into gossip protocols’ behavior in distributed cloud-native systems, evaluating aspects of scalability, reliability, and observability. | 1 | Abstract |
| Additional Result Tables :-  Table 5 - Bandwidth Networking Adjustment Observability.  Table 6. Memory Adjustment Observability.  Table 7. Different Location Observability. | 27 - 28 | Result |
| Additional Results Comments:-  Observability framework implemented in this study reveals critical configuration trade-offs in gossip protocol behavior under different configurations.   - Bandwidth Adjustment (Table 5): Increasing bandwidth allocation from 5 Mbps to 30 Mbps generally reduces CPU usage, particularly at higher node counts, suggesting that higher bandwidth allows for more efficient message processing. However, this efficiency comes at the cost of increased memory consumption, likely due to the need to handle larger data transfers. Transmission and reception volumes show complex patterns, indicating that bandwidth alone doesn't directly correlate with these metrics. - Memory Adjustment (Table 6): Increasing memory allocation from 150 Mi to 300 Mi results in improved CPU efficiency, likely by providing more resources for the gossip process. Memory usage remains comparable, indicating that the higher allocation is utilized effectively. Transmission and reception volumes show little correlation with memory allocation. - Location Observability (Table 7): Zonal deployments exhibit lower memory usage compared to regional setups, potentially due to reduced data replication overhead. However, zonal deployments show higher CPU usage, possibly due to increased communication overhead within the zone. Regional deployments, on the other hand, scale more effectively beyond 200 nodes, implying better performance for larger networks. | 15 - 16 | Result |
| Additional Discussions:-   - Observability Insights   The observability metric results highlight significant configuration trade-offs in gossip protocol performance. Increasing bandwidth reduces CPU usage but increases memory consumption, while larger memory allocations improve CPU efficiency with minimal impact on memory footprint. Deployment topology also plays a crucial role: zonal deployments offer memory savings but incur higher CPU demands, whereas regional setups scale more effectively for larger networks. In essence, resource scaling is non-linear and configuration-dependent, with memory usage showing more predictable trends compared to CPU usage and network traffic. Achieving optimal performance requires careful workload-aware tuning to balance network throughput, resource allocation, and deployment architecture. | 16 - 17 | Discussion |
| Analysis and Conclusion Amendment  Original:  This study presents a detailed examination of gossip protocols within cloud-native environments, significantly contributing to cloud-native computing and distributed systems. The research highlights the challenges with the scalability and performance of gossip protocols as the network size increases, providing a nuanced understanding of message propagation dynamics across varying network scales. This aspect is particularly significant for cloud-native systems, emphasizing the need for adaptable and scalable communication mechanisms to handle rapid network size changes without compromising efficiency.  The study also provides valuable insights into optimization strategies for implementing gossip protocols in cloud-native applications. By presenting concrete metrics on the impact of node quantity on protocol performance, the research validates theoretical expectations regarding scalability challenges. It proposes practical solutions for enhancing system reliability and message propagation speed. The findings of this research are highly relevant for industries that rely on robust, scalable, and efficient communication mechanisms, such as blockchain, IoT, and mobile applications. The study suggests tailored adjustments to gossip protocol configurations can significantly improve performance across diverse cloud-native settings.  Moreover, the study emphasizes gossip protocols' broader applicability and potential to advance distributed systems' resilience, scalability, and efficiency. By elucidating the protocol's dynamics within cloud-native environments, the research opens opportunities for more adaptable and robust distributed architectures, offering substantial theoretical and practical advancements. Consequently, this study not only enriches the existing body of knowledge on gossip protocols and cloud-native computing but also sets the stage for future innovations in deploying and optimizing distributed systems across various sectors.  Amended:  This study contributes to understanding gossip protocols in cloud-native environments by empirically validating theoretical assumptions and revealing infrastructure-specific behaviors. While some scalability patterns confirm established knowledge (like propagation delays at scale), the research demonstrates how elastic, pay-as-you-go cloud platforms fundamentally alter protocol dynamics - notably showing that 30Mbps bandwidth reduces CPU usage by 40% while increasing memory consumption by 15-20%, and that 300Mi memory allocations achieve 25% greater CPU efficiency than traditional 150Mi configurations. These quantified trade-offs, observable only in cloud- native, provide actionable optimization guidelines for blockchain and IoT deployments. The work establishes three key advances:  • a cloud-native methodology capturing multidimensional metrics (CPU, memory, network) simultaneously, overcoming limitations of conventional simulators like PeerSim;  • identified configuration thresholds that enable practical performance tuning; and  • a reproducible framework for future research on advanced factors like topology-aware propagation without dedicated hardware costs.  The significance of these findings lies in their practical applicability and methodological innovation. By bridging theoretical protocol behavior with cloud-native constraints, the research demonstrates that expected patterns manifest differently in elastic infrastructures - for instance, the observed 40% CPU reduction at 30Mbps incurs memory costs unique to dynamic environments. This work provides practitioners with evidence-based strategies for optimizing distributed systems, while offering researchers a cost-effective experimental platform to investigate scalability-resource trade-offs. The results not only advance gossip protocol implementation in production environments but also establish foundational knowledge for developing more resilient and adaptive cloud-native architectures. | 19 - 20 | Contributions and Significance of the Study |

**Comment 3:** The data availability statement indicates that data is available upon request through the author's email. Making the data accessible would enhance the study's credibility and allow independent validation of the findings.

**Author response:** We sincerely thank you for the valuable feedback regarding data availability. We agree that making the data publicly accessible enhances the study's credibility and allows for independent validation of the findings.

In response to this comment, we have updated the Data Availability Statement in the manuscript to reflect that the dataset is now publicly available. The dataset, including all relevant simulation data and analysis scripts, has been uploaded to a public repository on GitHub and accessed at the following URL: <https://github.com/wwiras/cnsim.git>

This repository includes:

- Source Data: All data used for the simulations, modeling, and analysis are presented in the manuscript.
- Documentation: Detailed instructions for accessing, reproducing, and validating the results.
- License: An open-source license to facilitate reuse and further research by the community.

This change aligns with best practices for open science and significantly improves the transparency and reproducibility of our work. Thank you for your suggestion, which has strengthened the manuscript.

The amendments are as follows:

| **Amendment** | **Page** | **Section** |
| --- | --- | --- |
| Original :  The steps taken to implement a gossip protocol in a cloud-native simulation are as follows:-  Amended:  The following steps outline the gossip protocol implementation for this cloud-native simulation. All research artifacts—including synthetically generated datasets and the complete source code—are publicly available at https://github.com/wwiras/cnsim.git. This work complies with all relevant ethical and legal guidelines, and no external restrictions apply to its data or methodology. | 12 | Steps for Implementing Gossip Protocol in a Cloud Native Simulation |

**End**
